# Healthcare utilisation and mortality in people with osteoarthritis in the UK: findings from a national primary care database

**DOI:** 10.3399/BJGP.2022.0419

**Published:** 2023-07-11

**Authors:** Subhashisa Swain, Carol Coupland, Aliya Sarmanova, Chang Fu Kuo, Christian Mallen, Michael Doherty, Weiya Zhang

**Affiliations:** Nuffield Department of Primary Care Health Sciences, University of Oxford, Oxford, UK; research associate (honorary), School of Medicine, University of Nottingham, Nottingham, UK.; School of Medicine, University of Nottingham, Nottingham, UK.; Bristol Medical School, University of Bristol, Bristol, UK.; Division of Rheumatology, Allergy and Immunology, Chang Gung Memorial Hospital, Taoyuan City, Taiwan.; School of Medicine, Keele University, Keele, UK.; School of Medicine, University of Nottingham; Pain Centre Versus Arthritis, University of Nottingham, Nottingham, UK.; School of Medicine, University of Nottingham; Pain Centre Versus Arthritis, University of Nottingham, Nottingham, UK.

**Keywords:** epidemiology, orthopaedics, osteoarthritis, primary health care, rheumatology

## Abstract

**Background:**

The burden of osteoarthritis (OA) in UK primary care has not been investigated thoroughly.

**Aim:**

To estimate healthcare use and mortality in people with OA (overall and joint specific).

**Design and setting:**

A matched cohort study of adults with an incident diagnosis of OA in primary care were selected for the study using UK national Clinical Practice Research Datalink (CPRD) electronic records.

**Method:**

Healthcare utilisation was measured as the annual average number of primary care consultations and admissions to hospital after the index date for any cause and all-cause mortality data in 221 807 people with OA and an equal number of controls (with no OA diagnosis) who were matched to the case patients by age (standard deviation 2 years), sex, practice, and year of registration. The associations between OA and healthcare utilisation and all-cause mortality were estimated using multinomial logistic regression and Cox regression, respectively, adjusting for covariates.

**Results:**

The mean age of the study population was 61 years and 58% were female. In the OA group, the median number of primary care consultations per year after the index date was 10.91 compared with 9.43 in the non-OA control group (*P* = 0.001) OA was associated with an increased risk of GP consultation and admission to hospital. The adjusted hazard ratio for all-cause mortality was 1.89 (95% confidence interval [CI] = 1.85 to 1.93) for any OA, 2.09 (95% CI = 2.01 to 2.19) for knee OA, 2.08 (95% CI = 1.95 to 2.21) for hip OA, and 1.80 (95% CI = 1.58 to 2.06) for wrist/hand OA, compared with the respective non-OA control group.

**Conclusion:**

People with OA had increased rates of GP consultations, admissions to hospital, and all-cause mortality that varied across joint sites.

## INTRODUCTION

Osteoarthritis (OA) is the most common chronic joint condition, affecting different sites and presenting with pain, functional impairment, and reduced quality of life.^1^^,^^2^ In recent decades, one in 10 people attending UK primary care consulted for OA.^3^ Globally, years of life with disability for hip and knee OA increased by 6.6 million over the period 1990 to 2010 (10.5 million in 1990 to 17.1 million in 2010).^4^ The rising prevalence of chronic conditions, such as OA, increases the burden on the health system, especially in primary care settings where most of these conditions are managed. OA incurs a large expenditure in primary care and is a financial burden to health systems worldwide.^5^^,^^6^ In addition to clinical need, healthcare utilisation in primary care depends on a wide range of factors such as socioeconomic and demographic factors, accessibility, and availability.^7^ There are various methods used to measure healthcare utilisation from a health system perspective. Two commonly used indicators are the number of hospital visits and the number of inpatient admissions per person.^8^ Previous studies have shown increased healthcare visits by people with knee OA compared with controls,^9^^,^^10^ although data for healthcare utilisation for joint-specific OA are currently lacking.

Another measure of disease burden is the risk of mortality. Traditionally, OA has been considered a low priority among chronic conditions because of a minimal risk of mortality despite it being highly prevalent and a significant cause of morbidity in older people.^11^ The evidence of an association between all-cause mortality and OA is inconclusive.^12^ Studies have shown significant associations with cause-specific cardiovascular disease (CVD) and all-cause mortality.^13^^,^^14^

In the context of multiple chronic conditions, it is not well understood whether the health utilisation pattern increases after the first diagnosis of OA. It is also important to study the outcomes for different types of OA based on the joint involved, as different joint involvement can have different physiological and pathological explanations, and result in different outcomes for those affected. Information on healthcare utilisation and mortality would provide a clearer picture of health resources used and can guide prioritisation in primary care. Therefore, the current study examined all-cause mortality, general practice consultations, and inpatient admissions in people with OA and matched controls using a large primary care database in the UK.

**Table:** How this fits in

Nearly 10% of the people attending primary care in the UK have osteoarthritis (OA) but their healthcare utilisation pattern in the health system is not well known. This study found people with OA had more primary care consultations and admissions to hospital, and increased all-cause mortality compared with similar age- and sex-matched controls. The burden was high for people diagnosed with hip, knee, and ankle/foot OA.

## METHOD

A matched retrospective cohort study was undertaken using Clinical Practice Research Datalink (CPRD) GOLD, which is the UK’s large prospective primary care electronic medical record database.^15^ The study involved analyses of anonymised patient-level data of ∼17.5 million individuals from 736 general practices as of 31 December 2017, generalisable to the wider UK population.^16^ It was approved by the Independent Scientific Advisory Committee for Medicines and Healthcare products Regulatory Agency (MHRA) database research (protocol number: 19_030R).

### Case definition of OA

Read codes, which are a standard clinical coding system used in UK general practice, were used to identify people with a diagnosis of incident OA between 1 January 1997 and 31 December 2017. For this study, the date of the first recorded physician diagnosis for hip, knee, ankle/foot, wrist/hand OA, or site recorded as ‘unspecified’ OA was used as the index date and the start date of follow-up. Other inclusion criteria were:

- aged ≥20 years at the index date; and
- having active registration for ≥36 months with an up-to-standard (UTS) practice (determined by CPRD database standards) before the index date.

Details of the selection of OA population are given in Supplementary Information S1.

### Selection of the control group

The control group was made up of people who were registered for ≥36 months with UTS practices and with no record of diagnosed OA, OA-related joint pain, or total joint replacement during the study period. One control participant per OA case patient matched by year of birth (standard deviation [SD] 2 years), sex, year of first registration, and practice was selected and assigned with the same index date as their matched case patient.

### Outcomes

#### Average number of annual primary care consultations

The definition of ‘consultation’ includes a consultation with a GP, practice nurse, or any other primary healthcare practitioner for any purpose that has been recorded in the CPRD GOLD database. The average was calculated by dividing the total number of GP consultations recorded per person from the index date until the last record available for the person or 31 December 2017, if earlier, by the number of years followed up. For example, if a person had a total of 15 years of follow-up and had 120 consultations recorded during that period, then the average annual number of consultations for that person was eight consultations per year (120/15).

#### Average number of annual inpatient admissions

Information on inpatient admissions was available for 432 572 people (97.5% of total study population) obtained from hospital episode statistics linkage data. The total number of admissions to hospital during follow-up, irrespective of cause, was divided by the years of follow-up to estimate average inpatient admissions per year for each person.

#### All-cause mortality

All-cause mortality data were obtained from Office for National Statistics linkage data. The recorded date of death was used in the analysis to estimate the mortality rate in the OA group compared with that in the non-OA group.

### Covariates

Information available at the index date including age, sex, smoking status, alcohol use, and body mass index (BMI) was used in the analysis. BMI (kg/m^2^) was categorised as underweight (<18.5), normal (18.5–24.9), overweight (25.0–29.9), obese (≥30.0), and missing. Smoking status was categorised as ex-smoker, current smoker, non-smoker, and missing. Alcohol use was grouped into non-user, ex-user, current user 1–9 units/week, current user ≥10 units/week, current user (unknown quantity), and missing.

The study also assessed the status of 49 chronic conditions at the index date in both groups, and the Elixhauser Comorbidity Index (ECI) at baseline was calculated to estimate the burden of comorbidities.^17^ Each comorbidity was categorised as either present or not at the index date. Details of the selection of chronic conditions and list of conditions are given in Supplementary Information S1 and Supplementary Table S1, respectively.

### Statistical analysis

The outcomes were compared between the OA and the matched control groups. Descriptive statistics for each outcome were reported as either mean (SD) or median (interquartile range [IQR]) as per distribution. Normal distribution of outcomes was checked using histograms and the Shapiro–Wilk test. Primary care consultations were grouped into four equal groups using quartiles, that is, 25% of participants per group. Hospital admissions were grouped into four unequal groups as about 60% of participants had zero admissions to hospital; the four groups were formed as ‘no admissions to hospital (zero)’ and three other groups using terciles.

Associations between consultation/admission to hospital groups and OA (yes/no) were analysed using the multinomial logistic regression model and reported as odds ratios (OR) and 95% confidence intervals (CIs). In the adjusted model, covariates included age, sex, smoking, alcohol, BMI, number of chronic conditions, and ECI at the index date. The ordinal logistic regression model was used to calculate the OR (95% CI) per quartile and *P*-values for trend.

The follow-up period for all-cause mortality was from the index date until the earliest date of death, transfer out of practice, or end of the study (31 December 2017). The Kaplan–Meier method was used to display the cumulative probability of all-cause mortality. Hazard ratios (HRs) and 95% CI were calculated using a Cox regression model adjusting for age, sex, BMI, smoking, alcohol use, count of chronic conditions, and ECI at the index date. The proportional hazard assumption was examined with Schoenfeld residual tests. In a sensitivity analysis, all-cause mortality risk was estimated in people with OA (*n* = 22 333) and age- (SD 2), sex-, registration-year-, and practice-matched controls (*n* = 22 333) without any of the 49 specified chronic conditions at baseline using the same approach as that for the full cohort.

The statistical analyses were performed using Stata (version 15) and R (version 3.5).

## RESULTS

A total of 221 807 OA case patients and 221 807 age-, sex-, registration-year-, and practice-matched non-OA control participants were included in the analysis (Table 1). Mean age of the study population was 61.05 years (SD 13.17) and 57.67% were female. Among people with OA, 71.51% had at least one record of unspecified OA and 24.72% had knee OA either alone or with other OA. The median number of chronic conditions in people with OA at the index date was 2 (IQR 1–4) compared with 1 (IQR 0–3) in the non-OA control group (*P*<0.05).

**Table 1.** Characteristics of incident OA group and matched control group at index date

**Characteristic**	**Incident OA group (*n* = 221 807)**	**Control group (*n* = 221 807)**	**Unadjusted odds ratio (95% CI)^a^**
**Total, age, mean (SD)**	61.05 (13.17)	60.88 (13.31)	N/A

**Male, age, mean (SD)**	60.71 (12.85)	60.54 (12.97)	N/A

**Female, age, mean (SD)**	61.30 (13.40)	61.12 (13.55)	N/A

**Age, years, *n* (%)**			
<40	12 266 (5.53)	13 018 (5.87)	N/A
40–49	30 809 (13.89)	31 673 (14.28)	N/A
50–59	60 287 (27.18)	59 606 (26.87)	N/A
60–69	60 442 (27.25)	59 924 (27.02)	N/A
70–79	40 879 (18.43)	40 418 (18.22)	N/A
80–89	15 926 (7.18)	15 815 (7.13)	N/A
≥90	1198 (0.54)	1353 (0.61)	N/A

**Sex, *n* (%)**			
Male	93 895 (42.33)	93 895 (42.33)	N/A
Female	127 912 (57.67)	127 912 (57.67)	N/A

**BMI (kg/m^2^)**			
BMI, mean (SD)	28.28 (5.62)	26.62 (4.98)	N/A
<18.5 (underweight), *n* (%)	3020 (1.36)	4738 (2.14)	0.85 (0.82 to 0.90)^b^
18.5–24.9 (normal), *n* (%)	63 531 (28.64)	85 534 (38.56)	Reference
25.0–29.9 (overweight), *n* (%)	82 683 (37.28)	82 190 (37.05)	1.35 (1.33 to 1.37)^b^
≥30 (obese), *n* (%)	72 442 (32.66)	46 898 (21.14)	2.08 (2.04 to 2.11)^b^
Missing, *n* (%)	131 (0.06)	2447 (1.10)	0.07 (0.06 to 0.09)^b^

**Alcohol consumption (units/week), *n* (%)**			
Never	44 084 (19.87)	40 889 (18.43)	Reference
Ex-drinker	6040 (2.72)	5311 (2.39)	1.05 (1.01 to 1.08)^b^
Current 1–9	77 549 (34.96)	79 502 (35.84)	0.89 (0.88 to 0.91)^b^
Current ≥10	43 148 (19.45)	42 753 (19.27)	0.93 (0.91 to 0.95)^b^
Current unknown	50 860 (22.93)	50 922 (22.96)	0.92 (0.91 to 0.94)^b^
Missing	126 (0.06)	2430 (1.10)	0.05 (0.04 to 0.06)^b^

**Smoking status, *n* (%)**			
Never smoked	117 498 (52.97)	122 551 (55.25)	Reference
Ex-smoker	41 683 (18.79)	39 646 (17.87)	1.15 (1.14 to 1.17)^b^
Current smoker	62 545 (28.20)	57 208 (25.79)	1.10 (1.08 to 1.12)^b^
Missing	81 (0.04)	2402 (1.08)	0.03 (0.02 to 0.04)^b^

**Joints involved, *n* (%)**			
Hip	25 091 (11.31)	N/A	N/A
Knee	54 841 (24.72)	N/A	N/A
Wrist/hand	13 255 (5.98)	N/A	N/A
Ankle/foot	5311 (2.39)	N/A	N/A
Unspecified	158 620 (71.51)	N/A	N/A

**ECI at index, mean (SD)**	62.74 (3.45)	62.51 (3.10)	N/A

**Number of chronic conditions at index, mean (SD)**	2.51 (2.14)	1.92 (1.89)	N/A

**Number of chronic conditions at index, median (IQR)**	2 (1–4)	1(0–3)	N/A

a *Matched by sex, age, practice, and index date.*

b P*< 0.05. BMI = body mass index. ECI = Elixhauser Comorbidity Index.*

*IQR = interquartile range. OA = osteoarthritis. OR = odds ratio. NA = not applicable. SD = standard deviation.*

### Primary care consultations

The median number of annual primary care consultations after the index date was higher in the OA group compared with the control group with medians of 10.91 (IQR 4.66–21.96) and 9.43 (IQR 3.64–20.35) (*P*<0.05), respectively (Table 2). Within the OA group, the median number of annual primary care consultations was 12.38 (IQR 5.59–24.31) for hip OA, 12.15 (IQR 5.34–23.93) for knee OA, and 11.66 (IQR 5.11–23.56) for ankle/foot OA.

**Table 2.** Summary for the average number of primary care consultations and hospital admissions per year in the OA and non-OA groups^a^

**Group**	**Primary care consultations per year after index date**		**Hospital admissions per year after index date**	

	**Median (IQR)**	**Mean (SD)**	**Median (IQR)**	**Mean (SD)**
OA (*n* = 221 807)	10.91 (4.66–21.96)	16.86 (20.04)	0 (0–0.19)	0.25 (1.70)

Non-OA (*n* = 221 807)	9.43 (3.64–20.35)	16.13 (21.22)	0 (0–0.09)	0.18 (1.09)

**Site of OA**				
Knee (*n* = 54 841)	12.15 (5.34–23.93)	18.45 (21.98)	0 (0–0.20)	0.25 (1.38)
Hip (*n* = 25 091)	12.38 (5.59–24.31)	18.72 (21.97)	0 (0–0.22)	0.25 (1.58)
Ankle/foot (*n* = 5311)	11.66 (5.11–23.56)	18.12 (22.16)	0 (0–0.17)	0.23 (0.67)
Wrist/hand (*n* = 13 255)	10.46 (4.46–20.86)	16.28 (20.18)	0 (0–0.14)	0.19 (0.68)
Unspecified (*n* = 158 620)	11.55 (5.02–22.79)	17.48 (20.40)	0 (0–0.19)	0.27 (1.93)

a *See Supplementary Table S3 for an expanded version of this table. IQR = interquartile range. OA = osteoarthritis. SD = standard deviation.*

**Figure 1.**
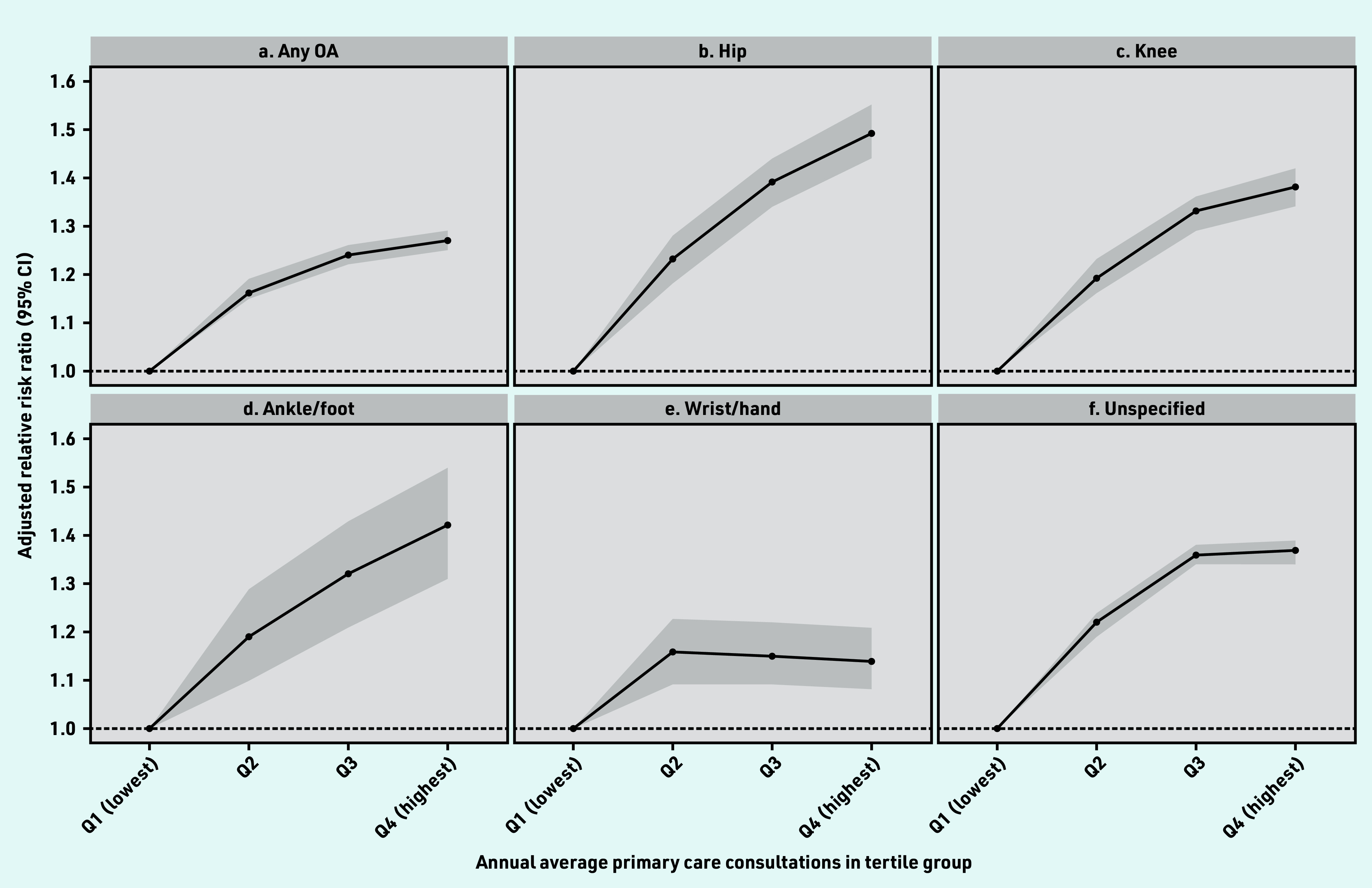
*Adjusted risk association with annual average primary care consultations in people with OA compared with non-OA. Adjusted for age, sex, smoking, alcohol, BMI, Elixhauser Comorbidity Index, and number of comorbidities at baseline using multinomial regression model. a) Any OA; b) hip; c) knee; d) ankle/foot; e) wrist/hand; and f) unspecified. Detailed information in Supplementary Table S4. Median and IQR values of average annual primary care consultations in each group: Q1, 1.84 (IQR 0.89–2.95); Q2, 6.84 (IQR 5.43–8.41); Q3, 14.61 (IQR 12.21–17.50); Q4, 33.66 (IQR 26.17–48.08). BMI = body mass index. IQR = interquartile range. OA = osteoarthritis. Q = quartile.*

The median number of annual consultations increased with increasing age in both sexes (Supplementary Figure S1). The OR for average annual consultations increased gradually from 1 for quartile 1 (referent) to 1.16 (95% CI = 1.15 to 1.19) for quartile 2, 1.24 (95% CI = 1.22 to 1.26) for quartile 3, and 1.27 (95% CI = 1.25 to 1.29) for quartile 4 (*P* for trend = 0.001) in the adjusted model (Figure 1a).

The association of the primary care consultations with quartile 4 in people with hip OA was 49% (OR 1.49, 95% CI = 1.44 to 1.55) more compared with quartile 1. The association was 42% higher for ankle/foot OA (OR 1.42, 95% CI = 1.31 to 1.54), and 38% more for knee OA (OR 1.38, 95% CI = 1.34 to 1.42) (Figures 1b–1e).

### Hospital admissions

In total, 65.00% (*n* = 144 174) of people in the OA group and 70.00% (*n* = 155 264) in the non-OA group had zero admissions to hospital after the index date during follow-up (data not shown). The median number of admissions to hospital per year increased with increasing age in both sexes and was higher in the OA group compared with the non-OA control group (Table 2 and Supplementary Figure S2).

**Figure 2.**
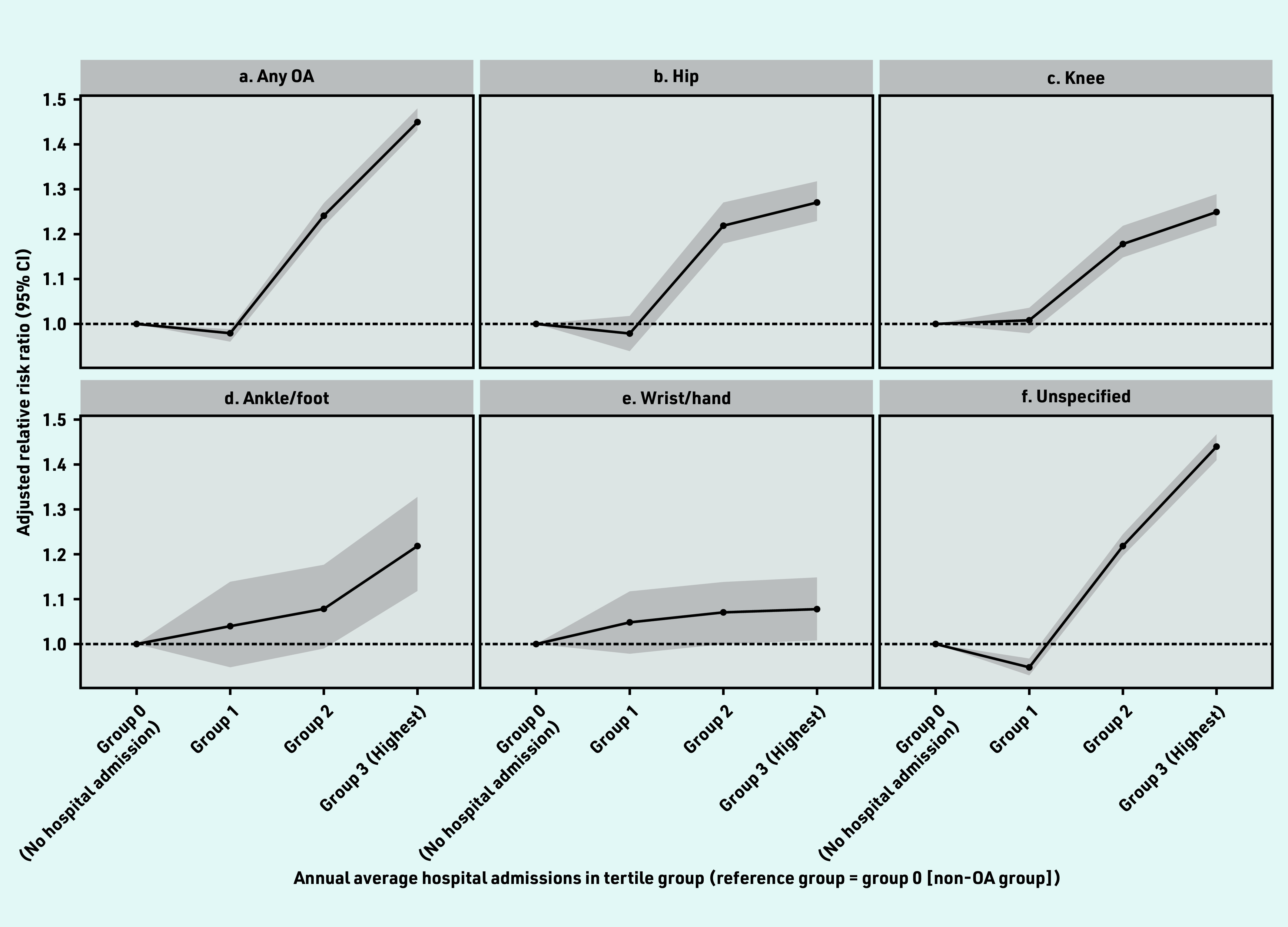
*Association with annual average number of hospital admissions per year in the OA and non-OA groups. Adjusted for age, sex, smoking, alcohol, BMI, Elixhauser Comorbidity Index, and number of chronic conditions at baseline. a) Any OA; b) hip; c) knee; d) ankle/foot; e) wrist/hand; and f) unspecified. Detailed information can be found in Supplementary Table S5. Median and IQR values of annual average admissions to hospital in each group: Group 0 — no hospital admissions. Group 1 — Q1, 0.11 (IQR 0.06–0.15); Group 2 — Q2, 0.32 (IQR 0.26–0.42); Group 3 — Q3, 0.97 (IQR 0.70–1.59). BMI = body mass index. IQR = interquartile range. OA = osteoarthritis. Q = quartile.*

After the index date, people with OA had a greater risk of hospital admissions than people without OA and the OR increased from 1 for zero admissions to hospital (referent) to 0.98 (95% CI = 0.96 to 1.00) for tertile 1, 1.24 (95% CI = 1.22 to 1.27) for tertile 2, and 1.46 (95% CI = 1.43 to 1.48) for tertile 3, respectively, in the adjusted model (Figure 2a). The association of unspecified, hip, and knee OA in the highest hospital admission group was 44% (OR 1.44, 95% CI = 1.41 to 1.47), 27% (OR 1.27, 95% CI = 1.23 to 1.33), and 25% (OR 1.25, 95% CI = 1.21 to 1.29) compared with the zero hospital admissions group, respectively (Figures 2b, 2c, and 2f).

### All-cause mortality

Of those with OA, 20 617 (9.3%) died during the study follow-up period, compared with 13 087 (5.9%) in the non-OA group. Median duration of follow-up in people with OA was 6.03 years (IQR 2.89–10.13) compared with 7.90 years (IQR 4.13–12.13) in the control group (data not shown). The crude all-cause mortality rate was nearly two times higher in the OA group (13.52 per 1000 person–years compared with 7.14 per 1000 person–years in the non-OA group). The adjusted HR comparing the OA group with the non-OA group was 1.89 (95% CI = 1.85 to 1.93) (Table 3).

**Table 3.** Association with all-cause mortality in the OA and non-OA groups

**Group**	**Total, *n***	**Deaths, *n***	**Mortality rate per 1000 person–years (95% CI)**	**Unadjusted HR (95% CI)**	**Adjusted HR (95% CI)^a^**
Non-OA control	221 807	13 087	7.14 (7.02 to 7.27)	Reference	Reference

OA (any site)	221 807	20 617	13.52 (13.34 to 13.70)	2.02 (1.98 to 2.06)^b^	1.89 (1.85 to 1.93)^b^

**Site of OA**					
Non-knee controls	59 351	3637	7.94 (7.68 to 8.20)	Reference	Reference
Knee	53 982	5038	14.70 (14.30 to 15.11)	1.98 (1.90 to 2.06)^b^	2.09 (2.01 to 2.19)^b^
Non-hip controls	27 521	1783	8.38 (8.00 to 8.78)	Reference	Reference
Hip	24 701	2503	15.90 (15.29 to 16.54)	2.03 (1.91 to 2.16)^b^	2.08 (1.95 to 2.21)^b^
Non-ankle/foot controls	5874	252	5.34 (4.72 to 6.04)	Reference	Reference
Ankle/foot	5231	355	9.89 (8.91 to 10.98)	1.95 (1.66 to 2.28)^b^	2.00 (1.70 to 2.36)^b^
Non-wrist/hand controls	9570	361	4.99 (4.50 to 5.53)	Reference	Reference
Wrist/hand	9729	605	9.68 (8.94 to 10.48)	2.03 (1.79 to 2.32)^b^	1.80 (1.58 to 2.06)^b^
Non-unspecified controls	161 568	10 055	7.43 (7.28 to 7.57)	Reference	Reference
Unspecified	155 540	14 981	14.27 (14.05 to 14.50)	2.05 (2.00 to 2.10)^b^	1.80 (1.75 to 1.84)^b^

a *Adjusted for age, sex, smoking, alcohol, BMI, ECI, and number of comorbidities at baseline.*

b P*<0.05. BMI = body mass index. ECI = Elixhauser Comorbidity Index. HR = hazard ratio. OA = osteoarthritis.*

**Figure 3.**
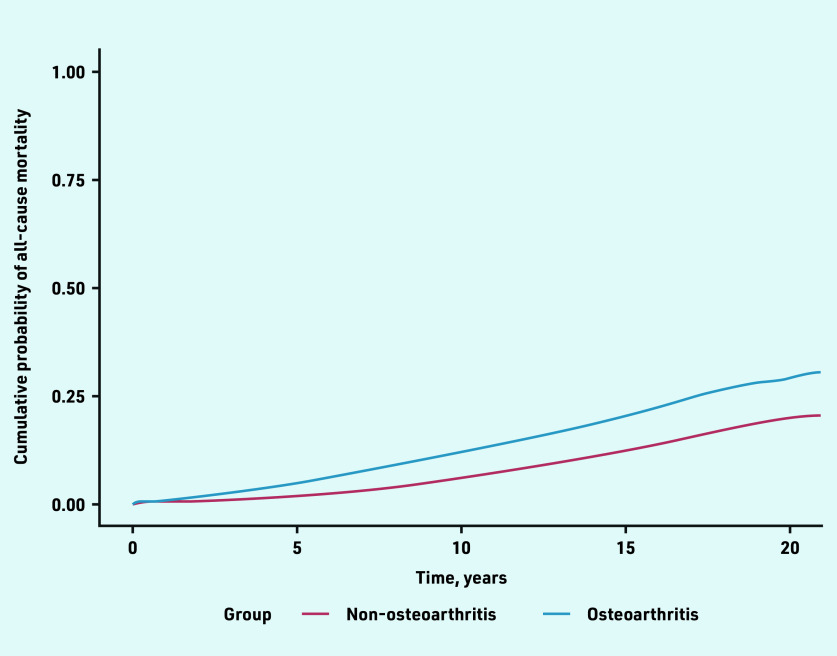
*Cumulative probabilities of all-cause mortality in the OA and non-OA groups after index date. OA = osteoarthritis.*

Knee OA (HR 2.09, 95% CI = 2.01 to 2.19) and hip OA (HR 2.08, 95% CI = 1.95 to 2.21) had higher risk of mortality followed by wrist/hand OA (HR 1.80, 95% CI = 1.58 to 2.06) (Table 3). Proportional hazard assumptions were satisfied. The cumulative probability of death increased with follow-up time and was higher in people with OA compared with people without OA (Figure 3). Joint-specific cumulative probability of mortality is provided in Supplementary Figures S3a–S3d.

### Sensitivity analysis

In the restricted matched cohorts without any of the 49 comorbidities at the index date, the mortality rate in the OA group was 6.26 per 1000 person–years compared with 2.99 in the non-OA control group. The HR for all-cause mortality in OA was 2.15 (95% CI = 2.00 to 2.43) after adjustment for other covariates (Supplementary Table S2). The cumulative probability of death was higher in people with OA (Supplementary Figure S4).

## DISCUSSION

### Summary

This study has demonstrated:

- people with OA had an increased number of GP consultations and admissions to hospital;
- people with OA had twice the mortality rate compared with people without OA; and
- the associations varied slightly between joint sites and were independent from age, sex, BMI, and comorbidities.

### Strengths and limitations

Major strengths of the study are the inclusion of 49 comorbidities, a long follow-up, and adjusting for the number and severity of other chronic conditions in analysis of outcomes.

Several limitations include the following: ascertainment biases because of misdiagnosis, miscoding, and delayed recording in the GP database; many people had unspecified OA, which weakens the findings regarding site-specific OA; and only all-cause mortality was estimated, whereas cause-specific mortality might provide further insights about specific causal pathways to the excess deaths in people with OA.

There is underreporting of OA in primary care because of ‘joint replacement’ in secondary care.^18^ The hospital admissions and primary care visits were calculated irrespective of any specific reason, which could have been influenced by the diagnosis of other conditions. People who visited more frequently may have had more chance of being diagnosed with multiple chronic conditions; however, both the number and severity of the chronic conditions at the index date were adjusted for. Differences in lifestyle and health behaviour patterns and medication use were not considered, which might have confounded the associations with OA.

### Comparison with existing literature

The reasons for the increased consultation rate per year in the OA group is multifactorial. Musculoskeletal problems are the second highest reason for consultations in UK primary care.^19^ It is most likely to result from joint pain or incident comorbidities.^20^^,^^21^ For OA, frequent visits are to confirm the diagnosis through further clinical examinations such as radiographs, and/or are solely for management of pain and disability because of OA.^20^ Bedson *et al* found no difference in the median number of consultations for comorbidities between people who consulted for knee pain and those who did not consult for knee pain.^20^ In the current study, the number of chronic conditions at the index date were adjusted for and an association was still found with higher consultation rates in people with OA. Often people living with more chronic conditions are prescribed with multiple medications, thus demanding more visits for medication review in primary care. Surprisingly, the current study found the annual average primary care consultation rate after the index date increased in people with ankle/foot OA, one of the least researched sites for OA compared with the knee.^9^^,^^22^ The increased consultation rate overall shows the high burden for people with OA using primary care services. As these consultations could be for OA or other factors such as medications, this merits more detailed investigation and comparison with other chronic conditions.

Average annual hospital admissions were higher in people with OA, similar to that reported in the US^22^ and the UK.^23^ The number and burden of other chronic conditions in those with OA has been suggested as the cause of increased admissions to hospital;^24^ however, the findings from the current study with the model adjusted for the ECI index and count of chronic conditions at baseline suggests the excess in hospital admissions could be because of new comorbidities developing after the diagnosis of OA. Increased risks of falls and injury^25^ and the requirement for joint replacement, especially of the knee,^26^^,^^27^ could be the reasons for these rates of hospital admissions.

Another important associated factor could be adverse events, such as gastrointestinal bleeding^28^ and cardiovascular disease (CVD), from use of analgesics, such as non-steroidal anti-inflammatory drugs (NSAIDs), in OA.^29^ The association of unspecified OA sites with increased annual average hospital admissions after the index date is difficult to explain in the absence of a clear definition of unspecified OA. The non-significant association with wrist/hand OA indirectly suggests less secondary healthcare resource use.

The evidence for an association of all-cause mortality with OA is inconclusive.^14^^,^^30^ The current study found people with OA had an excess all-cause mortality rate compared with the non-OA control group, similar to that reported in the Somerset and Avon Survey of Health study.^13^ Reasons for these discordant findings may be as a result of methodological differences, including the definition of OA, age range, study design, and length of follow-up. Several reasons, apart from comorbidities, may help explain the higher mortality in people with OA, for example, obesity, pain, and disability or functional limitations.^13^ Another explanation could be the risk of CVD from chronic subclinical inflammation^31^ or the use of analgesics such as NSAIDs.^32^ In this study, comorbidity counts and the severity of comorbidities at the index date along with BMI at the index date were adjusted for in the model, but they did not explain the increased mortality rate. In the sensitivity analysis people did not have any comorbidities at the index date — the increased mortality rate could be as a result of the subsequent higher comorbidity incidence in the OA group after the index date rather than pre-existing comorbidities at the time of OA diagnosis. Cause-specific mortality was not part of the current research; further studies to explore the causes and pattern of mortality appear warranted.

### Implications for research and practice

People with OA have significantly increased annual primary care consultations and admissions to hospital, and double the all-cause mortality rate in the UK, compared with people without OA. It would be interesting to find out the exact factors associated with increase in hospitalisation, to reduce the burden on health services. Such data would provide essential information to estimate additional costs incurred in individuals with OA and the cost-effectiveness of a specific intervention. The increasing burden of OA healthcare utilisation in primary care should be noted and the reasons should be identified in order to design effective strategies to reduce the burden.

## Data

The authors used anonymised data on individual patients on which the analysis, results, and conclusions reported in the article are based. The Clinical Practice Research Datalink data are not distributed under licence. However, the relevant data can be obtained directly from the agency (https://www.cprd.com). The codes developed for the analysis are available on valid request.

## References

[1] Abhishek A, Doherty M (2013). Diagnosis and clinical presentation of osteoarthritis. Rheum Dis Clin North Am.

[2] Abbott JH, Usiskin IM, Wilson R (2017). The quality-of-life burden of knee osteoarthritis in New Zealand adults: a model-based evaluation. PLOS ONE.

[3] Swain S, Sarmanova A, Mallen C (2020). Trends in incidence and prevalence of osteoarthritis in the United Kingdom: findings from the Clinical Practice Research Datalink (CPRD). Osteoarthritis Cartilage.

[4] Cross M, Smith E, Hoy D (2014). The global burden of hip and knee osteoarthritis: estimates from the global burden of disease 2010 study. Ann Rheum Dis.

[5] Xie F, Kovic B, Jin X (2016). Economic and humanistic burden of osteoarthritis: a systematic review of large sample studies. PharmacoEconomics.

[6] Chen A, Gupte C, Akhtar K (2012). The global economic cost of osteoarthritis: how the UK compares. Arthritis.

[7] Costa D, Rodrigues AM, Cruz EB (2021). Driving factors for the utilisation of healthcare services by people with osteoarthritis in Portugal: results from a nationwide population-based study. BMC Health Serv Res.

[8] Andersen R, Newman JF (1973). Societal and individual determinants of medical care utilization in the United States. Milbank Mem Fund Q Health Soc.

[9] Kiadaliri A, Englund M (2021). Trajectory of excess healthcare consultations, medication use, and work disability in newly diagnosed knee osteoarthritis: a matched longitudinal register-based study. Osteoarthritis Cartilage.

[10] Peat G, McCarney R, Croft P (2001). Knee pain and osteoarthritis in older adults: a review of community burden and current use of primary health care. Ann Rheum Dis.

[11] Garin N, Koyanagi A, Chatterji S (2016). Global multimorbidity patterns: a cross-sectional, population-based, multi-country study. J Gerontol A Biol Sci Med Sci.

[12] Hochberg MC (2008). Mortality in osteoarthritis. Clin Exp Rheumatol.

[13] Nuesch E, Dieppe P, Reichenbach S (2011). All cause and disease specific mortality in patients with knee or hip osteoarthritis: population based cohort study. BMJ.

[14] Cleveland RJ, Nelson AE, Callahan LF (2019). Knee and hip osteoarthritis as predictors of premature death: a review of the evidence. Clin Exp Rheumatol.

[15] Herrett E, Gallagher AM, Bhaskaran K (2015). Data resource profile: Clinical Practice Research Datalink (CPRD). Int J Epidemiol.

[16] Herrett E, Thomas SL, Schoonen WM (2010). Validation and validity of diagnoses in the General Practice Research Database: a systematic review. Br J Clin Pharmacol.

[17] van Walraven C, Austin PC, Jennings A (2009). A Modification of the Elixhauser comorbidity measures into a point system for hospital death using administrative data. Med Care.

[18] Yu D, Jordan KP, Peat G (2018). Underrecording of osteoarthritis in United Kingdom primary care electronic health record data. Clin Epidemiol.

[19] Versus Arthritis (2023). The state of musculoskeletal health 2023: arthritis and other musculoskeletal conditions in numbers. https://www.versusarthritis.org/media/25613/versus-arthritis-state-msk-musculoskeletal-health-2023.pdf.

[20] Bedson J, Mottram S, Thomas E, Peat G (2007). Knee pain and osteoarthritis in the general population: what influences patients to consult?. Fam Pract.

[21] Swain S, Coupland C, Mallen C (2021). Temporal relationship between osteoarthritis and comorbidities: a combined case control and cohort study in the UK primary care setting. Rheumatology (Oxford).

[22] Wright EA, Katz JN, Cisternas MG (2010). Impact of knee osteoarthritis on health care resource utilization in a US population-based national sample. Med Care.

[23] Morgan OJ, Hillstrom HJ, Ellis SJ (2019). Osteoarthritis in England: incidence trends from National Health Service Hospital Episode Statistics. ACR Open Rheumatol.

[24] Palladino R, Lee JT, Ashworth M (2016). Associations between multimorbidity, healthcare utilisation and health status: evidence from 16 European countries. Age Ageing.

[25] Dore A, Golightly YM, Mercer V (2013). Risk of falls increases with additional symptomatic osteoarthritic joints: the Johnston County Osteoarthritis Project. https://acrabstracts.org/abstract/risk-of-falls-increases-with-additional-symptomatic-osteoarthritic-joints-the-johnston-county-osteoarthritis-project.

[26] Culliford D, Maskell J, Judge A (2015). Future projections of total hip and knee arthroplasty in the UK: results from the UK Clinical Practice Research Datalink. Osteoarthritis Cartilage.

[27] Ackerman IN, Bohensky MA, Zomer E (2019). The projected burden of primary total knee and hip replacement for osteoarthritis in Australia to the year 2030. BMC Musculoskelet Disord.

[28] Henry D, Lim LLY, Rodriguez LAG (1996). Variability in risk of gastrointestinal complications with individual non-steroidal anti-inflammatory drugs: results of a collaborative meta-analysis. BMJ.

[29] Jüni P, Nartey L, Reichenbach S (2004). Risk of cardiovascular events and rofecoxib: cumulative meta-analysis. Lancet.

[30] Turkiewicz A, Neogi T, Björk J (2016). All-cause mortality in knee and hip osteoarthritis and rheumatoid arthritis. Epidemiology.

[31] Couzin-Frankel J (2010). Inflammation bares a dark side. Science.

[32] Trelle S, Reichenbach S, Wandel S (2011). Cardiovascular safety of non-steroidal anti-inflammatory drugs: network meta-analysis. BMJ.

